# Association Between Electronic Health Record Time and Quality of Care Metrics in Primary Care

**DOI:** 10.1001/jamanetworkopen.2022.37086

**Published:** 2022-10-18

**Authors:** Lisa S. Rotenstein, A. Jay Holmgren, Michael J. Healey, Daniel M. Horn, David Y. Ting, Stuart Lipsitz, Hojjat Salmasian, Richard Gitomer, David W. Bates

**Affiliations:** 1 Division of General Internal Medicine, Department of Medicine, Brigham and Women's Hospital, Boston, Massachusetts; 2 Harvard Medical School, Boston, Massachusetts; 3 Division of Hospital Medicine, Department of Medicine, University of California at San Francisco; 4 Division of General Internal Medicine, Department of Medicine, Massachusetts General Hospital, Boston; 5 Department of Pediatrics, Massachusetts General Hospital, Boston; 6 Department of Epidemiology, Harvard School of Public Health, Boston, Massachusetts

## Abstract

**Importance:**

Physicians across the US spend substantial time working in the electronic health record (EHR), with primary care physicians (PCPs) spending the most time. The association between EHR time and ambulatory care quality outcomes is unclear.

**Objective:**

To characterize measures of EHR use and ambulatory care quality performance among PCPs.

**Design, Setting, and Participants:**

A cross-sectional study of PCPs with longitudinal patient panels using a single EHR vendor was conducted at Brigham and Women’s Hospital and Massachusetts General Hospital during calendar year 2021.

**Exposures:**

Independent variables included PCPs demographic and practice characteristics and EHR time measures (PCP-level mean of daily total EHR time, after-hours time, time from 5:30 pm to 7:00 am and time on weekends, and daily EHR time on notes, sending and receiving patient, staff, results, prescription, or system messages [in-basket], and clinical review).

**Main Outcomes and Measures:**

Outcome variables were ambulatory quality measures (year-end, PCP panel–level achievement of targets for hemoglobin A_1c_ level control, lipid management, hypertension control, diabetes screening, and breast cancer screening).

**Results:**

The sample included 291 physicians (174 [59.8%] women). Median panel size was 829 (IQR, 476-1157) patients and mean (SD) clinical full-time equivalent was 0.54 (0.27). The PCPs spent a mean (SD) of 145.9 (64.6) daily minutes on the EHR. There were significant associations between EHR time and panel-level achievement of hemoglobin A_1c_ control, hypertension control, and breast cancer screening targets. In adjusted analyses, each additional 15 minutes of total daily EHR time was associated with 0.58 (95% CI, 0.32-0.84) percentage point greater panel-level hemoglobin A_1c_ control, 0.52 (95% CI, 0.33-0.71) percentage point greater hypertension control, and 0.28 (95% CI, 0.05-0.52) higher breast cancer screening rates. Each daily additional 15 minutes of in-basket time was associated with 2.26 (95% CI, 1.05-3.48) greater panel-wide hemoglobin A_1c_ control, 1.65 (95% CI, 0.83-2.47) percentage point greater hypertension control, and 1.26 (95% CI, 0.51-2.02) percentage point higher breast cancer screening rates. Associations were largely concentrated among PCPs with 0.5 clinical full-time equivalent or less. There were no associations between EHR use metrics and diabetes screening or lipid management in patients with cardiovascular disease.

**Conclusions and Relevance:**

This cross-sectional study found an association between EHR time and some measures of ambulatory care quality. Although increased EHR time is associated with burnout, it may represent a level of thoroughness or communication that enhances certain outcomes. It may be useful for future studies to characterize payment models, workflows, and technologies that enable high-quality ambulatory care delivery while minimizing EHR burden.

## Key Points

**Question:**

Is there an association between measures of electronic health record (EHR) use and ambulatory quality performance among primary care physicians?

**Findings:**

In this cross-sectional study of 291 primary care physicians, there were significant associations between multiple electronic health record time measures and panel-level achievement of hemoglobin A_1c_ control, hypertension control, and breast cancer–screening targets. For example, each additional 15 minutes of daily time spent on EHR messaging was associated with 2.3% greater panel-wide hemoglobin A_1c_ level control, 1.7% greater hypertension control, and 1.3% higher breast cancer screening rates.

**Meaning:**

The findings of this study suggest that increased electronic health record time may represent a level of thoroughness or communication that enhances quality outcomes.

## Teaser

This cross-sectional study assesses the association of primary care physician time spent on electronic health record (EHR) tasks and 5 common measure of adult ambulatory care quality.

## Introduction

Despite associations shown between broad electronic health record (EHR) adoption and information exchange and safety,^1,2,3^ increased use of the EHR has substantially changed the work patterns of physicians in the US. Physicians across the US are spending substantial time on the EHR, and notably more than their non-US counterparts.^4^ However, the association of this time with quality of care they deliver is unclear.

Ambulatory care quality measures, such as those endorsed by the National Committee for Quality Assurance, range from metrics evaluating chronic care processes and outcomes to those related to provision of preventive care such as mammography and vaccination.^5^ In addition to serving as a means of comparing care delivery across systems and setting a standard for processes and outcomes, ambulatory care quality measures are frequently used to determine compensation in traditional payer contracts and alternative payment arrangements.^6,7,8^ Given the outpatient, chronic care, and preventive nature of many of these metrics, primary care physicians (PCPs) and their teams often bear substantial responsibility for ambulatory care quality measure performance.

Primary care physicians also spend the most time on the EHR among ambulatory care specialties.^9^ Specifically, they spend more total and after-hours time on the EHR and receive many more clinical messages than their medical and surgical specialist counterparts.^9^ Increased time working in the EHR, particularly after hours, has been associated with burnout.^10,11^ Although in some studies burnout is associated with lower quality of care^12^ and self-reported medical errors,^1^ there is other evidence to suggest that physicians who report burnout may have better ambulatory care outcomes. Specifically, in a study of family physicians, Casalino et al^13^ noted that physicians who reported some frequency of burnout had generally lower rates of ambulatory care–sensitive admissions, ambulatory care–sensitive emergency department visits, and hospital readmissions.

Given that EHR burden is a potential factor associated with burnout in PCPs, understanding the associations between EHR time and outcomes could help disentangle the evidence on the association between burnout and quality. On the one hand, it is possible that physicians who spend more time on the EHR, for example, on panel or clinical review, may be more conscientious and this characteristic might also be associated with improved ambulatory care quality outcomes. On the other hand, additional daily time on the EHR or notes may signal a burden of EHR-related work or documentation that prohibits sufficient focus on disease management or between-visit follow-up, which could adversely influence ambulatory quality outcomes. Time spent in the EHR may also have differential outcomes depending on where that time is spent. For example, documentation time may be less important for improving outcomes compared with time spent messaging with a patient to help manage their care, and EHR time after hours may mean that a PCP spends more time face-to-face with their patients during clinic hours. Furthermore, the association between EHR time and quality outcomes may vary by a PCP’s clinical workload; the ability to message patients electronically may be more valuable to physicians spending less time in clinical practice and more time on research or administrative duties.

Despite the burden of the EHR for PCPs and their heavy involvement in ambulatory care quality, the associations between diverse measures of EHR use and ambulatory care quality performance have not been characterized. To address this question, we conducted a cross-sectional study examining the association between physician-level EHR use data and achievement of ambulatory care quality metrics across 2 academic medical center primary care networks during 2021. We had 3 main research questions: What is the association between overall time spent in the EHR and performance across ambulatory quality measures for primary care physicians? How does that association vary across time spent in different functions of the EHR? How do these associations differ between physicians with different levels of clinical workload, as measured by the proportion of their time dedicated to patient care? Understanding these associations is a critical step for identifying where and how EHRs can improve primary care quality.

## Methods

### Sample

Our sample included all 338 practicing attending PCPs at Brigham and Women’s Hospital (BWH) and Massachusetts General Hospital (MGH). These 2 academic medical centers, which have primary care practice networks consisting of 18 (BWH) and 17 (MGH) primary care practices that are part of the Mass General Brigham system. Both practices implemented the same instance of the same EHR vendor system (EpicCare, Epic Systems), use similar panel design and time allocation structures, and have adopted the same system-wide slate of quality metrics.^14^ The practices are mainly off-site, in the community, and they included 6 community health centers. We focused our analyses on the 316 attending PCPs with a longitudinal panel because panel-level ambulatory care quality metrics are only calculated for physicians who see patients longitudinally. Resident PCPs were not included in this analysis. The study was deemed exempt by the BWH Institutional Review Board owing to its focus on secondary data analysis. This study followed the Strengthening the Reporting of Observational Studies in Epidemiology (STROBE) reporting guideline.

We extracted demographic and practice data, Epic Signal EHR (the EHR system) use metadata that includes metrics of EHR use, and ambulatory care quality metric data for all PCPs who had a patient panel for all of calendar year 2021. We excluded 25 PCPs who established their practice between 2019 and 2021, because the first 3 years in practice are considered a ramp-up period within the Mass General Brigham system during which PCPs are meeting and establishing their patient panels. Our final analytic sample thus consisted of 291 PCPs.

### Demographic and Practice Data

For each PCP, we collected data on the hospital with which their primary care practice was affiliated (BWH or MGH), their sex (male or female), their panel size, the proportion of their full time equivalent (FTE) that they practiced clinical medicine (hereafter referred to as clinical FTE), their mean panel risk score (based on a Health and Human Services–derived risk score for adults with commercial insurance that incorporates information about age and hierarchical condition categories^15^), and the number of years since their completion of residency. Consistently and accurately collected race and ethnicity data were not available and thus not analyzed. In the Mass General Brigham system, PCP attribution is determined by the name in the PCP field in the EHR. This is entered on scheduling of the initial appointment or arrival to the initial appointment depending on the practice. Target panel size is adjusted for clinical FTE after an initial ramp-up period. Thus, panel size and clinical FTE were assumed to be collinear in our analysis.

### EHR Data

We extracted EHR use data for all PCPs between January and December 2021. By averaging values from each of 12-monthly the EHR system data extracts, we calculated physician-level daily means of multiple EHR use metrics.^4^ Specifically, for each PCP, we calculated means of total daily EHR time, time outside of scheduled hours (defined as any active time that falls outside a 30-minute buffer before or after a physician’s first and final visits on a scheduled day), pajama time (defined as 5:30 pm to 7:00 am and time on weekends), time on composing notes, time spent in the in-basket (Epic’s module for sending and receiving PCP, staff, and system messages), time on clinical review (eg, review of notes, test results, and other clinical information), and time spent placing orders. Although there may be overlap between time outside of scheduled hours and pajama time, time outside of scheduled hours may also occur during the workday hours when clinical care is not scheduled. All EHR system time metrics are based on active time, which is defined as the time a user is performing active tasks.^16^ If no activity is detected for 5 seconds, the system stops counting time. This measurement captures EHR interaction while excluding time a clinician may spend with the EHR open but performing other activities. Epic additionally provides an estimate of the percentage of orders placed by a physician that have a team contribution, and we calculated mean values for this metric for each PCP over the 12-month period.

### Ambulatory Quality Outcomes

For our outcome variables, we focused on 5 adult ambulatory care quality metrics that are most likely to be influenced by physicians, are well accepted among PCPs, and less likely to be affected by scheduling lags: hemoglobin A_1c_ level control in established diabetes, lipid management in established cardiovascular disease, blood pressure control in patients with established hypertension, screening for diabetes, and breast cancer screening. These metrics are determined based on national quality standards and payer contracts and are standardized across the Mass General Brigham system.^14^ Detailed metric definitions can be found in the eAppendix in the Supplement.

The data elements necessary to calculate the numerator and denominator for each quality measure are extracted from the EHR. Meeting the standard is defined as a laboratory value or vital sign at the appropriate level, or the presence of a completed imaging procedure. The PCPs receive point-of-care decision support in the EHR for unmet measures, as well as monthly reports of overall performance. We used 2021 year-end performance on these ambulatory quality metrics in our analyses, because year-end values are used in system-wide performance assessments, practice and system-wide initiatives may be targeted at achieving performance targets by year-end, and year-end performance would be the most likely to be associated with PCP time spent on the EHR across the calendar year. All variables are the proportion of patients in a PCPs’ panel that achieved the quality standard for that measure, expressed as a percentage between 0 and 100%.

### Statistical Analysis

We descriptively analyzed demographic characteristics, practice, quality, and EHR use patterns for all PCPs in the study sample. We additionally stratified these analyses by whether PCPs had a greater than 0.5 clinical FTE.

Next, we built ordinary least-squares multivariable linear regression models examining the association between each quality metric as the dependent variable and specific EHR use metrics (total daily time, time outside scheduled hours, pajama time, time on notes, time on clinical review, in-basket time) as independent variables, adjusting for PCP sex, clinical FTE, mean panel risk score, whether the PCP practice is a health center, and hospital (BWH vs MGH. Given a less clear hypothetical association between time on orders and quality outcomes, we did not include this metric as an independent variable in regression analyses. In sensitivity analyses, we additionally adjusted for each PCPs’ years postresidency. We ran sensitivity analyses using quantile regression methods given the ability of this method to evaluate the association between 2 variables across a wider range of outcome variable values. We used the Benjamini-Hochberg false discovery rate to account for multiple comparisons in each quality score grouping for both unadjusted and adjusted analyses for the main outcome.

To identify whether associations between quality and EHR time were different for PCPs with different amounts of clinical FTE and panel sizes, we conducted 2 sets of stratified analyses. First, we stratified multivariable analyses of EHR use vs quality by whether a PCP’s clinical FTE was greater than 0.5. As a sensitivity analysis, we subsequently stratified multivariable EHR use vs quality analyses by panel size (below or above mean for sample) rather than clinical FTE. Once again, we used the Benjamini-Hochberg false discovery rate to account for multiple comparisons in each quality score grouping.

All analyses were conducted using SAS OnDemand for Academics, 2021 (SAS Institute Inc), with a 2-sided significance threshold of *P* = .05.

## Results

We included a total of 291 physicians (92.0% of all PCPs with a longitudinal panel) in our analyses (Table 1). Of all physicians, 174 were women (59.8%) and 117 were men (40.2%). Median panel size was 829 (IQR, 476-1157) patients and their mean (SD) FTE was 0.54 (0.27). Mean (SD) panel risk scores were 1.93 (0.48). These values were similar across physicians with more than 0.5 clinical FTE vs with less than or equal to 0.5 clinical FTE.

**Table 1.** PCP Demographic Characteristics

Characteristic	No. (%)		
	Total (n = 291)	Clinical FTE ≤0.5 (n = 158)	Clinical FTE >0.5 (n = 133)
Hospital			
Brigham and Women’s Hospital	130 (44.7)	72 (45.6)	58 (43.6)
Massachusetts General Hospital	161 (55.3)	86 (54.4)	75 (56.4)
Sex			
Women	174 (59.8)	94 (59.5)	80 (60.2)
Men	117 (40.2)	64 (40.5)	53 (39.8)
Clinical FTE, mean (SD)	0.54 (0.27)	0.34 (0.14)	0.78 (0.17)
Panel Risk Score, mean (SD)	1.93 (0.48)	2.02 (0.51)	1.83 (0.43)
Panel size, mean (SD), No. of patients	901.9 (542.0)	572.9 (318.0)	1292.7 (446.6)
Proportion practicing at a community health center	63 (21.6)	33 (20.9)	30 (22.6)

Abbreviations: FTE, full-time equivalent; PCP, primary care physician.

As reported in Table 2, PCPs spent a mean (SD) of 145.9 (64.6) daily minutes on the EHR in total. They spent a mean (SD) of 70.0 (43.2) minutes outside of scheduled hours, 60.7 (57.0) minutes of pajama time per day, 42.9 (30.7) minutes daily on notes, 31.0 (13.9) minutes daily on the in-basket, and 26.1 (13.6) minutes daily on clinical review. Across our sample, a mean (SD) of 6.4 (7.8) of PCPs’ orders had a team contribution. The mean time in each of these categories was greater for PCPs with a greater than 0.5 clinical FTE.

**Table 2.** PCP EHR Use Characteristics

EHR time categories	Mean (SD), min		
	Total (n = 291)	Clinical FTE ≤0.5 (n = 158)	Clinical FTE >0.5 (n = 133)
Total daily time	145.5 (64.6)	110.2 (54.3)	187.4 (49.2)
Daily time outside scheduled hours	70.0 (43.2)	65.9 (42.8)	74.9 (43.3)
Pajama time	60.7 (57.0)	59.2 (60.4)	62.4 (52.9)
Daily time on notes	42.9 (30.7)	32.0 (24.4)	55.9 (32.3)
Daily in-basket time	31.0 (13.9)	25.7 (13.7)	37.4 (11.3)
Daily clinical review time	26.1 (13.6)	20.2 (11.2)	33.1 (13.0)
Daily order time	25.8 (15.4)	18.3 (11.3)	34.8 (14.8)
% Of orders with team contribution	6.4 (7.8)	6.0 (6.4)	6.8 (4.9)

Abbreviations: EHR, electronic health record; FTE, full-time equivalent; PCP, primary care physician.

The mean (SD) performance on quality metrics was 76.9% (8.3%) for hemoglobin A_1c_ control in patients with diabetes, 79.0% (6.8%) for blood pressure control, 84.2 (7.0%) for lipid control in those with established cardiovascular disease, 93.4% (3.9%) for diabetes screening, and 80.7% (7.9%) for breast cancer screening (Table 3). These values were similar across physicians with more than 0.5 clinical FTE vs with less than or equal to 0.5 clinical FTE.

**Table 3.** Distribution of Percent of PCPs’ Panel Meeting Each Quality Metric Target

Quality metric	% Of eligible patients on panel meeting metric target, mean (SD)		
	Total (n = 291)	Clinical FTE <0.5 (n = 158)	Clinical FTE ≥0.5 (n = 133)
HbA_1c_ control in established diabetes	76.9 (8.3)	77.7 (9.3)	76.0 (6.9)
Blood pressure control in patients with essential hypertension	79.0 (6.8)	79.6 (6.9)	78.3 (6.5)
Breast cancer screening	80.7 (7.9)	81.2 (8.5)	80.1 (7.0)
Lipid management in established CVD	84.2 (7.0)	84.6 (7.4)	83.8 (6.6)
Diabetes screening	93.4 (3.9)	93.0 (4.2)	93.7 (3.5)

Abbreviations: CVD, cardiovascular disease; FTE, full-time equivalent; HbA_1c_, hemoglobin A_1c_; PCP, primary care physician.

Both unadjusted and adjusted analyses revealed associations between PCPs’ EHR time and panel-level achievement of hemoglobin A_1c_ control, hypertension control, and breast cancer screening targets. As presented in Table 4, in adjusted analyses, each additional 15 minutes of total daily EHR time was associated with 0.58 (95% CI, 0.32-0.84) percentage point greater panel-wide hemoglobin A_1c_ control, 0.52 (95% CI, 0.33-0.71) percentage point greater panel-wide hypertension control, and 0.28 (95% CI, 0.05-0.52) percentage point higher breast cancer screening rates. Each additional 15 minutes of daily time outside scheduled hours was associated with 0.66 (95% CI, 0.31-1.00) percentage point greater panel-wide hemoglobin A_1c_ control, 0.60 (95% CI, 0.40-0.81) percentage point greater panel-wide hypertension control, and 0.37 (95% CI, 0.08-0.65) percentage point higher panel-wide breast cancer screening rates. Each additional 15 minutes of pajama time was associated with 0.19 (95% CI, 0.02-0.36) percentage point greater panel-wide breast cancer screening. The numeric association between each additional 15 minutes of pajama time and hypertension control was statistically significant but numerically minimal (0.003; 95% CI, 0.001-0.004 percentage point difference), and there was no association between additional pajama time and hemoglobin A_1c_ control.

**Table 4.** Adjusted Estimated Differences in PCPs’ Panel-Level Percent Achievement of Metric Targets per Each Additional 15 Minutes of Daily EHR Time (N = 291)^a^

EHR time category	Percentage of panel meeting metric target, β (95% CI)									
	HbA_1c_ control	*P* value^b^	Hypertension control	*P* value^b^	Breast cancer screening	*P* value^b^	Lipid management in established CVD	*P* value^b^	Diabetes screening	*P* value^b^
Total daily time	0.58 (0.32 to 0.84)	<.001	0.52 (0.33 to 0.71)	<.001	0.28 (0.05 to 0.52)	.03	0.14 (−0.10 to 0.38)	.54	0.004 (−0.15 to 0.15)	.96
Time outside scheduled hours	0.66 (0.31 to 1.00)	.001	0.60 (0.40 to 0.81)	<.001	0.37 (0.08 to 0.65)	.03	0.21 (−0.09 to 0.52)	.54	−0.06 (−0.21 to 0.09)	.72
Pajama time	0.24 (−0.02 to 0.50)	.07	0.003 (0.001 to 0.004)	.001	0.19 (0.02 to 0.36)	.03	0.03 (−0.24 to 0.30)	.83	−0.04 (−0.16 to 0.08)	.72
Clinical review time	1.64 (0.49 to 2.78)	.006	1.19 (0.41 to 1.98)	.003	−0.17 (−1.21 to 0.88)	.76	−0.28 (−1.18 to 0.62)	.81	0.22 (−0.36 to 0.80)	.72
Notes time	0.64 (0.21 to 1.07)	.006	0.58 (0.26 to 0.89)	.001	0.43 (0.02 to 0.83)	.048	0.06 (−0.45 to 0.58)	.83	−0.06 (−0.27 to 0.16)	.74
In-basket time	2.26 (1.05 to 3.48)	.001	1.65 (0.83 to 2.47)	<.001	1.26 (0.51 to 2.02)	.006	0.57 (−0.42 to 1.57)	.54	−0.27 (−0.99 to 0.45)	.72

Abbreviations: CVD, cardiovascular disease; HbA_1c_, hemoglobin A_1c_; EHR, electronic health record; FTE, full-time equivalent; PCP, primary care physician.

^a^ Models adjusted for PCPs’ sex, clinical FTE, mean panel risk score, and whether PCP practice is a health center, and hospital (Brigham and Women’s Hospital vs Massachusetts General Hospital). Standard errors are clustered by PCP practice site.

^b^ *P* value reflects adjustment for false discovery rate using Benjamini-Hochberg method.

There were also associations between EHR time spent on specific activities and hemoglobin A_1c_ control, hypertension control, and breast cancer screening (Table 4). In adjusted analyses, each additional 15 minutes of daily clinical review time was associated with 1.64 (95% CI, 0.49-2.78) percentage point greater panel-wide hemoglobin A_1c_ control and 1.19 (95% CI, 0.41-1.98) percentage point greater panel-wide hypertension control. There was no association between additional clinical review time and breast cancer screening rates. Each additional 15 minutes of daily time on notes was associated with 0.64 (95% CI, 0.21-1.07) percentage point greater panel-wide hemoglobin A_1c_ control, 0.58 (95% CI, 0.26-0.89) percentage point greater hypertension control, and 0.43 (95% CI, 0.02-0.83) percentage point higher panel-wide breast cancer screening rates. In addition, each 15 minutes of daily in-basket time was associated with 2.26 (95% CI, 1.05-3.48) percentage point greater panel-wide hemoglobin A_1c_ control, 1.65 (95% CI, 0.83-2.47) percentage point greater panel-wide hypertension control, and 1.26 (95% CI, 0.51-2.02) percentage point higher breast cancer screening rates. Associations noted persisted in models additional adjusting for PCPs’ years since residency (eTable 1 in the Supplement). Associations also persisted in models using quantile regression (eTable 2 in the Supplement); although findings for various EHR time categories and achievement of panel-level breast cancer screening metric were not significant at the median, the positive direction persisted. There were no associations between EHR use metrics and diabetes screening or lipid control in patients with established cardiovascular disease.

As reported in Table 5, associations between EHR time and panel-level quality metric performance were largely concentrated in PCPs with less than or equal to 0.5 clinical FTE. Among PCPs with less than or equal to 0.5 clinical FTE, each additional 15 minutes of total daily EHR time was associated with significantly greater panel-level hemoglobin A_1c_ control (0.75; 95% CI, 0.53-0.97 percentage points), hypertension control (0.48; 95% CI, 0.22-0.75 percentage points), and breast cancer screening (0.39; 95% CI, 0.15-0.63) percentage points; however, among PCPs with more than 0.5 clinical FTE, we noted associations only between hypertension control and several EHR time metrics. For example, each additional 15 minutes of daily EHR time was associated with 0.55 (95% CI, 0.27-0.84) percentage points greater hypertension control. Each additional 15 minutes of daily EHR time outside of scheduled hours were associated with 0.46 (95% CI, 0.18-0.74) percentage points greater hypertension control and 15 minutes of daily notes time were associated with 0.47 (95% CI, 0.10-0.84) percentage points greater hypertension control. Similarly, among PCPs with less than or equal to 0.5 clinical FTE, each additional 15 minutes of daily in-basket time was associated with a significantly greater level of hemoglobin A_1c_ control (2.84; 95% CI, 1.53-4.15 percentage points, hypertension control (1.93; 95% CI, 0.76-3.10 percentage points, and breast cancer screening (1.58; 95% CI, 0.53-2.63 percentage points); these findings were not significant among PCPs with greater than 0.5 clinical FTE. These patterns were similar in sensitivity analyses stratified by below- vs above-average panel size rather than clinical FTE (eTable 3 and eTable 4 in the Supplement).

**Table 5.** Adjusted Estimated Differences in PCPs’ Panel-Level Percent Achievement of Metric Targets per Each Additional 15 Minutes of Daily EHR Time, by PCP Clinical FTE^a^

EHR time category	Percentage of panel meeting metric target, β (95% CI)									
	HbA_1c_ control	*P* value^b^	Hypertension control	*P* value^b^	Breast cancer screening	*P* value^b^	Lipid management in established CVD	*P* value^b^	Diabetes screening	*P* value^b^
**PCPs with ≤0.5 clinical FTE (n = 158)**										
Total daily time	0.75 (0.53 to 0.97)	<.001	0.48 (0.22 to 0.75)	.001	0.39 (0.15 to 0.63)	.004	0.31 (−0.02 to 0.64)	.16	−0.04 (−0.31 to 0.22)	.91
Time outside scheduled hours	0.89 (0.54 to 1.24)	<.001	0.72 (0.46 to 0.98)	<.001	0.53 (0.13 to 0.93)	.01	0.32 (−0.11 to 0.75)	.23	−0.13 (−0.37 to 0.10)	.81
Pajama time	0.33 (0.02 to 0.63)	.048	0.003 (0.001 to 0.004)	.001	0.26 (0.03 to 0.48)	.04	0.12 (−0.14 to 0.38)	.43	−0.02 (−0.23 to 0.19)	.91
Clinical review time	3.26 (1.80 to 4.71)	<.001	1.62 (0.38 to 2.87)	.01	0.48 (−1.00 to 1.96)	.52	−0.10 (−2.05 to 1.85)	.93	0.06 (−0.99 to 1.12)	.91
Notes time	1.28 (0.74 to 1.82)	<.001	0.88 (0.36 to 1.40)	<.001	0.86 (0.46 to 1.27)	<.001	0.54 (−0.06 to 1.15)	.16	−0.09 (−0.50 to 0.32)	.91
In-basket time	2.84 (1.53 to 4.15)	<.001	1.93 (0.76 to 3.10)	.002	1.58 (0.53 to 2.63)	.007	1.51 (0.14 to 2.88)	.16	−0.73 (−1.96 to 0.50)	.81
**PCPs with >0.5 clinical FTE (n = 133)**										
Total daily time	0.29 (−0.08 to 0.67)	.36	0.55 (0.27 to 0.84)	.001	0.23 (−0.14 to 0.61)	.47	0.02 (−0.36 to 0.40)	.97	0.11 (−0.07 to 0.30)	.56
Time outside scheduled hours	0.35 (−0.12 to 0.83)	.36	0.46 (0.18 to 0.74)	.004	0.19 (−0.21 to 0.58)	.47	0.13 (−0.25 to 0.52)	.87	0.07 (−0.09 to 0.23)	.56
Pajama time	0.05 (−0.29 to 0.39)	.78	0.002 (−0.001 to 0.004)	.17	0.10 (−0.13 to 0.32)	.47	−0.17 (−0.64 to 0.31)	.87	−0.03 (−0.13 to 0.07)	.64
Clinical review time	0.38 (−0.72 to 1.48)	.60	1.04 (−0.005 to 2.08)	.06	−0.17 (−1.60 to 1.27)	.82	−0.45 (−1.44 to 0.54)	.87	0.49 (−0.11 to 1.09)	.56
Notes time	0.19 (−0.28 to 0.65)	.60	0.47 (0.10 to 0.84)	.02	0.23 (−0.25 to 0.71)	.47	−0.23 (−1.04 to 0.58)	.87	0.04 (−0.28 to 0.36)	.81
In-basket time	1.09 (−0.51 to 2.69)	.36	1.21 (−0.02 to 2.43)	.06	0.86 (−0.37 to 2.08)	.47	−0.03 (−1.47 to 1.41)	.97	0.32 (−0.29 to 0.94)	.56

Abbreviations: CVD, cardiovascular disease; EHR, electronic health record; FTE, full-time equivalent; HbA_1c_, hemoglobin A_1c_; PCP, primary care physician.

^a^ Models adjusted for PCPs’ sex, clinical FTE, mean panel risk score, and whether PCP practice is a health center, and hospital (Brigham and Women’s Hospital vs Massachusetts General Hospital). Standard errors are clustered by PCP practice site.

^b^ *P* value reflects adjustment for false discovery rate using Benjamini-Hochberg method.

## Discussion

We performed a cross-sectional study of PCPs across the primary care networks of 2 large academic medical centers and found an association between EHR time and several measures of ambulatory care quality. The magnitude of estimated differences in panel-level achievement of quality metrics per each additional 15 minutes spent on specific EHR activities is in the range of results previously reported for quality improvement and accountable care initiatives.^17,18^ Our findings suggest that although increased EHR time, particularly after hours, has been associated with increased emotional exhaustion and burnout,^10,11^ it may represent a level of thoroughness, attention to detail, or patient and team communication that ultimately enhances certain outcomes. This finding is consistent with recent research reporting a trend toward better outcomes for measures of health care use for family physicians who reported some level of burnout,^13^ suggesting that the extra attention given to clinical problems and extra communication that may occur during additional time spent by PCPs may be valuable for patient outcomes. These results underscore the need to create team structures, examine PCP and office workflows, and enhance EHR-based technologies and decision support tools in ways that enable high quality of care, while optimizing time spent on the EHR.

It is notable that among all metrics of EHR time examined, there was the greatest numerical association between daily time on the in-basket and daily time on clinical review and ambulatory quality outcomes. Although time spent on clinical review on the day of a visit can now be accounted and billed for under the 2021 Evaluation and Management coding changes,^19^ time spent on the in-basket is typically not compensated. Rather, in-basket work is performed in addition to visit-based, revenue-generating work, often outside of scheduled clinic hours. In addition to substantially increasing since the COVID-19 pandemic,^20^ time spent addressing in-basket content has been associated with an increased likelihood of burnout and intent to reduce clinical hours.^21^ Our findings suggest that as PCPs and patients have become more comfortable with electronic correspondence, PCPs who spend more time corresponding with their patients and team members about care coordination, test results, and clinical follow-up (eg, titration of blood pressure or diabetes medications) may be more likely to achieve target clinical outcomes for diabetes and hypertension and to have higher rates of breast cancer screening in their panels. The associations we have identified between increased in-basket time and enhanced ambulatory quality of care highlight the importance of continuing to develop and expand value-based reimbursement systems that adequately reward outside-of-visit care delivery. The identified associations also raise also raises the potential for bringing workflow processes and automation technologies to bear to assist PCPs in more efficiently handling their clinical messages. Future work may also benefit from identifying the specific types of in-basket messaging activities associated with improved outcomes.

Among our sample, associations between time spent on the EHR and achievement of quality metric targets were concentrated among PCPs with clinical FTEs of 0.5 or less and below-average panel sizes, despite similar quality performance between physicians with clinical FTEs above and below 0.5. In a Canadian study of more than 4000 PCPs, increasing PCP panel size was associated with decreases in cancer screening, continuity, and comprehensiveness, although there was not an association between panel size and chronic disease indicators.^22^ Among family physicians, increasing panel size was associated with decreased access to care and diabetes quality metrics.^23^ Our findings of associations between EHR time and quality being concentrated among PCPs with lower clinical FTEs and panel sizes build on this literature. The findings suggest that additional time spent on the EHR by PCPs with a greater clinical FTE may not as clearly translate into actions or workflows that augment measured ambulatory quality of care, perhaps because of the demands of these PCPs’ visit-based work. Our results showing the association between time spent in the in-basket and quality measures for part-time PCPs also suggest that these individuals may depend more on asynchronous work to advance population health. Future research should investigate the differential outcomes of EHR functions, such as automated outreach^24^ or team-based ambulatory quality interventions^25,26^ for physicians with part-time compared with full-time clinical schedules.

It is not certain why there were associations between EHR time metrics and quality outcomes for some quality metrics but not for others, such as lipid control in cardiovascular disease and diabetes screening. Given the high levels of appropriate diabetes screening (mean, 93.4%) across the panels of BWH and MGH PCPs it is possible that there was a ceiling effect and not sufficient variation in this metric to show an association with EHR time. Also, both BWH and MGH have population health teams that support PCPs in tracking their quality performance. Given that a binary action, such as prescription of a high-dose statin, can count as meeting the lipid control in the cardiovascular disease metric, it is possible that these system-level supports more heavily influence lipid control outcomes than individual PCP time spent.

### Limitations

This study has limitations. One of these is its focus on 2 academic medical centers whose PCPs and their practice may not be representative of the broader US PCP population. In addition, both BWH and MGH have established population health programs developed over several years, which likely influence ambulatory quality outcomes for all PCPs in our sample. With the current data sources, we were unable to examine PCP EHR use at the level of specific patient visits and interactions. In addition, given the lack of data available at the level of the patient-PCP interaction, we were unable to adjust for other factors, such as continuity of patients’ care, individual patients’ interactions with other staff (eg, clinical pharmacists), or the number of consultants involved in an individual patient’s care.

Balancing these limitations are several strengths. To our knowledge, our study is the first to pair quality-of-care data with granular measures of EHR use. In addition, we were able to leverage detailed information about characteristics of PCPs and their panels, which enabled adjustment for factors such as panel complexity that might influence the association between EHR time and quality.

## Conclusions

This study showed an association between time spent on the EHR by PCPs and multiple measures of ambulatory care quality. These analyses suggest that time spent on in-basket and clinical review may particularly influence quality outcomes, with benefits largely concentrated among PCPs with lower clinical FTEs and panel sizes. Future studies should seek to identify the specific work patterns that contribute to the associations we have identified and characterize payment strategies, workflows, and technologies that can facilitate PCPs delivering high-quality ambulatory care while minimizing EHR burden.

## Notes

Supplement. **eAppendix.** Ambulatory Quality Metric Target Definitions **eTable 1.** Adjusted Estimated Differences in PCPs’ Panel-Level Percent Achievement of Metric Targets Per Each Additional 15 Minutes of Daily EHR Time, Including Adjustment for Post-Residency Years **eTable 2.** Adjusted Estimated Differences in PCPs’ Panel-Level Percent Achievement of Metric Targets Per Each Additional 15 Minutes of Daily EHR Time, Based on Quantile Regression **eTable 3.** Adjusted Estimated Differences in PCPs’ Panel-Level Percent Achievement of Metric Targets Per Each Additional 15 Minutes of Daily EHR Time, for PCPs with Below-Average Panel Size (N=158) **eTable 4.** Adjusted Estimated Differences in PCPs’ Panel-Level Percent Achievement of Metric Targets Per Each Additional 15 Minutes of Daily EHR Time, for PCPs with Above-Average Panel Size (N=133)
