# Supplementary material for: Association Between Electronic Health Record Time and Quality of Care Metrics in Primary Care
Source: JAMA Netw Open. 2022 Oct 18;5(10):e2237086. doi: 10.1001/jamanetworkopen.2022.37086 (PMC9579903; doi:10.1001/jamanetworkopen.2022.37086)
Supplement: Supplement. — eAppendix. Ambulatory Quality Metric Target Definitions eTable 1. Adjusted Estimated Differences in PCPs’ Panel-Level Percent Achievement of Metric Targets Per Each Additional 15 Minutes of Daily EHR Time, Including Adjustment for Post-Residency Years eTable 2. Adjusted Estimated Differences in PCPs’ Panel-Level Percent Achievement of Metric Targets Per Each Additional 15 Minutes of Daily EHR Time, Based on Quantile Regression eTable 3. Adjusted Estimated Differences in PCPs’ Panel-Level Percent Achievement of Metric Targets Per Each Additional 15 Minutes of Daily EHR Time, for PCPs with Below-Average Panel Size (N=158) eTable 4. Adjusted Estimated Differences in PCPs’ Panel-Level Percent Achievement of Metric Targets Per Each Additional 15 Minutes of Daily EHR Time, for PCPs with Above-Average Panel Size (N=133) [file jamanetwopen-e2237086-s001.pdf]

Supplementary Online Content

Rotenstein LS, Holmgren AJ, Healey MJ, et al. Association between electronic health record time and quality of care metrics in primary care. *JAMA Netw Open*. 2022;5(10):e2237086. doi:10.1001/jamanetworkopen.2022.37086

eAppendix. Ambulatory Quality Metric Target Definitions

eTable 1. Adjusted Estimated Differences in PCPs' Panel-Level Percent Achievement of Metric Targets Per Each Additional 15 Minutes of Daily EHR Time, Including Adjustment for Post-Residency Years

eTable 2. Adjusted Estimated Differences in PCPs' Panel-Level Percent Achievement of Metric Targets Per Each Additional 15 Minutes of Daily EHR Time, Based on Quantile Regression

eTable 3. Adjusted Estimated Differences in PCPs' Panel-Level Percent Achievement of Metric Targets Per Each Additional 15 Minutes of Daily EHR Time, for PCPs with Below-Average Panel Size (N=158)

eTable 4. Adjusted Estimated Differences in PCPs' Panel-Level Percent Achievement of Metric Targets Per Each Additional 15 Minutes of Daily EHR Time, for PCPs with Above-Average Panel Size (N=133)

This supplementary material has been provided by the authors to give readers additional information about their work.

© 2022 Rotenstein LS et al. *JAMA Network Open.*

eAppendix. Ambulatory Quality Metric Target Definitions

- **Hemoglobin A1c -** A patient between the ages of 18 and 75 (inclusive) is counted as meeting the metric target if their most recent A1c recorded in the EHR in the last 12 months was < 7.0% or if the A1c recorded in the EHR within the last 6 months was ≤ 9.0%.
- **Hypertension Control -** A patient between the ages of 18 and 85 (inclusive) is counted as meeting the metric target if their blood pressure has been measured within the last 12 months and either 1) the average of their last three blood pressure readings was < 130/80 or 2) if their blood pressure has been measured within the past 6 months and their last reading or the last three readings meet one of the following criteria: ≤140/90 if age < 60 or age ≥ 60 with diabetes; ≤ 150/90 if age ≥ 60 without diabetes; or diastolic BP < 70 and age ≥ 60; patient is on three or more anti-hypertensive medications from three distinct classes.
- **Breast Cancer Screening-** Women between the ages of 50 and 74 or who are otherwise clinically indicated for a mammogram (as noted by a clinician in the breast cancer screening registry) are considered to meet the metric target if they receive a mammogram according to the frequency specified by their clinician in the screening registry (biannual is default).
- **Lipid Management in Established Cardiovascular Disease** - Adult patients (males between the ages of 21 to 75 and female patients between the ages of 40 to 75) are considered to meet the lipid control in established cardiovascular disease criteria if their direct or calculated LDL is < 100 or they are on a high-dose statin.
- **Diabetes Screening** - Patients between the ages of 40 to ≤70 with a BMI > 25 kg/m^2 who have not previously been diagnosed with diabetes are considered to meet the diabetes screening metric target if they have had at least one diabetes test (from among a fasting blood glucose, A1c, glucose tolerance test, or a random glucose ≤125) documented within the last 3 years.

© 2022 Rotenstein LS et al. *JAMA Network Open.*

eTable 1. Adjusted* Estimated Differences in PCPs' Panel-Level Percent Achievement of Metric Targets Per Each Additional 15 Minutes of Daily EHR Time, Including Adjustment for Post-Residency Years

**EHR Time Category** *All Relationships Shown Based on 15-minute Time Increments*	**Hemoglobin A1c Control** *(β (95% CI) for Percentage of Panel Meeting Metric Target)*	**p-value****	**Hypertension Control** *(β (95% CI) for Percentage of Panel Meeting Metric Target)*	**p-value****	**Breast Cancer Screening** *(β (95% CI) for Percentage of Panel Meeting Metric Target)*	**p-value****	**Lipid Management in Established CVD** *(β (95% CI) for Percentage of Panel Meeting Metric Target)*	**p-value****	**Diabetes Screening** *(β (95% CI) for Percentage of Panel Meeting Metric Target)*	**p-value****
Total Daily Time	0.66 (0.39 to 0.92)	<0.001	0.56 (0.35 to 0.76)	<0.001	0.32 (0.08 to 0.56)	0.02	0.26 (0.02 to 0.50)	0.12	0.06 (-0.10 to 0.23)	0.92
Time Outside Scheduled Hours	0.70 (0.33 to 1.07)	0.0004	0.62 (0.40 to 0.85)	<0.001	0.38 (0.09 to 0.68)	0.02	0.33 (0.06 to 0.59)	0.12	-0.01 (-0.18 to 0.16)	0.98
Pajama Time	0.27 (0.01 to 0.53)	0.04	0.003 (0.001 to 0.004)	0.001	0.21 (0.04 to 0.38)	0.02	0.14 (-0.05 to 0.33)	0.20	-0.05 (-0.18 to 0.07)	0.92
Clinical Review Time	1.83 (0.55 to 3.11)	0.006	1.22 (0.35 to 2.09)	0.003	-0.06 (-1.20 to 1.08)	0.92	0.02 (-0.91 to 0.96)	0.96	0.26 (-0.38 to 0.90)	0.92
Notes Time	0.80 (0.37 to 1.22)	0.0004	0.66 (0.33 to 0.99)	0.001	0.47 (0.05 to 0.90)	0.04	0.33 (-0.07 to 0.72)	0.20	0.06 (-0.22 to 0.35)	0.98
In Basket Time	2.38 (1.06 to 3.70)	0.0001	1.72 (0.85 to 2.58)	0.0002	1.35 (0.61 to 2.08)	0.0002	0.72 (-0.32 to 1.75)	0.20	-0.01 (-0.73 to 0.71)	0.98

***** Models adjust for PCPs' gender, clinical FTE, average panel risk score, whether PCP's practice is a health center, hospital (BWH vs. MGH), and years since residency. Standard errors are clustered by PCP practice site.

**p-value reflects adjustment for false discovery rate using Benjamini-Hochberg method

© 2022 Rotenstein LS et al. *JAMA Network Open.*

eTable 2. Adjusted* Estimated Differences in PCPs' Panel-Level Percent Achievement of Metric Targets Per Each Additional 15 Minutes of Daily EHR Time, based on Quantile Regression

**EHR Time Category**<br><br>*All Relationships Shown Based on 15-minute Time Increments*	**Hemoglobin A1c Control**<br><br>*(β (95% CI) for Percentage of Panel Meeting Metric Target)*	**p-value****	**Hypertension Control**<br><br>*(β (95% CI) for Percentage of Panel Meeting Metric Target)*	**p-value****	**Breast Cancer Screening**<br><br>*(β (95% CI) for Percentage of Panel Meeting Metric Target)*	**p-value****	**Lipid Management in Established CVD**<br><br>*(β (95% CI) for Percentage of Panel Meeting Metric Target)*	**p-value****	**Diabetes Screening**<br><br>*(β (95% CI) for Percentage of Panel Meeting Metric Target)*	**p-value****
Total Daily Time	0.57 (0.16 to 0.98)	0.02	0.44 (-0.22 to 0.67)	0.0003	0.15 (-0.15 to 0.45)	0.40	0.19 (-0.15 to 0.52)	0.52	0.09 (-0.06 to 0.24)	0.70
Time Outside Scheduled Hours	0.63 (0.16 to 1.11)	0.02	0.51 (0.31 to 0.70)	<0.001	0.22 (-0.13 to 0.57)	0.36	0.33 (-0.06 to 0.72)	0.52	0.09 (-0.10 to 0.27)	0.70
Pajama Time	0.05 (-0.25 to 0.35)	0.74	0.25 (0.05 to 0.46)	0.02	0.08 (-0.15 to 0.30)	0.50	0.12 (-0.16 to 0.40)	0.52	0.03 (-0.12 to 0.19)	0.78
Clinical Review Time	1.37 (-0.01 to 2.76)	0.08	1.11 (0.28 to 1.94)	0.01	-0.62 (-1.65 to 0.41)	0.36	-0.47 (-1.63 to 0.70)	0.52	0.43 (-0.13 to 0.99)	0.70
Notes Time	0.52 (-0.22 to 1.26)	0.20	0.54 (0.05 to 1.04)	0.03	0.34 (-0.19 to 0.87)	0.36	0.14 (-0.35 to 0.62)	0.58	0.11 (-0.19 to 0.40)	0.74
In Basket Time	1.97 (0.45 to 3.49)	0.02	1.82 (0.80 to 2.84)	0.001	0.87 (-0.26 to 1.99)	0.36	0.67 (-0.51 to 1.85)	0.52	0.09 (-0.53 to 0.71)	0.78

***** Models adjust for PCPs' gender, clinical FTE, average panel risk score, whether PCP's practice is a health center, and hospital (BWH vs. MGH). Standard errors are clustered by PCP practice site.

**p-value reflects adjustment for false discovery rate using Benjamini-Hochberg method

© 2022 Rotenstein LS et al. *JAMA Network Open.*

eTable 3. Adjusted* Estimated Differences in PCPs' Panel-Level Percent Achievement of Metric Targets Per Each Additional 15 Minutes of Daily EHR Time, for PCPs with Below-Average Panel Size (N=158)

**EHR Time Category** *All Relationships Shown Based on 15-minute Time Increments*	**Hemoglobin A1c Control** *(β (95% CI) for Percentage of Panel Meeting Metric Target)*	**p-value****	**Hypertension Control** *(β (95% CI) for Percentage of Panel Meeting Metric Target)*	**p-value****	**Breast Cancer Screening** *(β (95% CI) for Percentage of Panel Meeting Metric Target)*	**p-value****	**Lipid Management in Established CVD** *(β (95% CI) for Percentage of Panel Meeting Metric Target)*	**p-value****	**Diabetes Screening** *(β (95% CI) for Percentage of Panel Meeting Metric Target)*	**p-value****
Total Daily Time	0.59 (0.26 to 0.93)	0.001	0.53 (0.24 to 0.82)	0.0008	0.37 (0.07 to 0.68)	0.03	0.28 (-0.07 to 0.64)	0.24	0.12 (-0.14 to 0.39)	0.80
Time Outside Scheduled Hours	0.61 (0.18 to 1.05)	0.008	0.73 (0.50 to 0.96)	<0.001	0.54 (0.08 to 0.99)	0.03	0.37 (-0.10 to 0.83)	0.24	0.04 (-0.24 to 0.33)	0.80
Pajama Time	0.21 (-0.10 to 0.51)	0.18	0.003 (0.001 to 0.005)	0.001	0.24 (-0.01 to 0.50)	0.07	0.10 (-0.24 to 0.44)	0.61	-0.04 (-0.19 to 0.11)	0.80
Clinical Review Time	2.70 (1.21 to 4.19)	0.001	1.81 (0.56 to 3.07)	0.006	0.54 (-1.23 to 2.30)	0.55	0.40 (-1.11 to 1.90)	0.61	0.21 (-0.94 to 1.36)	0.80
Notes Time	0.82 (0.08 to 1.56)	0.04	0.92 (0.21 to 1.62)	0.01	0.79 (0.12 to 1.46)	0.03	0.27 (-0.71 to 1.26)	0.61	0.31 (-0.04 to 0.66)	0.54
In Basket Time	2.57 (1.25 to 3.88)	0.0006	1.98 (0.83 to 3.13)	0.001	1.59 (0.33 to 2.84)	0.03	1.41 (0.15 to 2.68)	0.18	-0.15 (-1.35 to 1.04)	0.80

***** Models adjust for PCPs' gender, clinical FTE, average panel risk score, whether PCP's practice is a health center, and hospital (BWH vs. MGH). Standard errors are clustered by PCP practice site.

**p-value reflects adjustment for false discovery rate using Benjamini-Hochberg method

© 2022 Rotenstein LS et al. *JAMA Network Open.*

eTable 4. Adjusted* Estimated Differences in PCPs' Panel-Level Percent Achievement of Metric Targets Per Each Additional 15 Minutes of Daily EHR Time, for PCPs with Above-Average Panel Size (N=133)

**EHR Time Category** *All Relationships Shown Based on 15-minute Time Increments*	**Hemoglobin A1c Control** *(β (95% CI) for Percentage of Panel Meeting Metric Target)*	**p-value****	**Hypertension Control** *(β (95% CI) for Percentage of Panel Meeting Metric Target)*	**p-value****	**Breast Cancer Screening** *(β (95% CI) for Percentage of Panel Meeting Metric Target)*	**p-value****	**Lipid Management in Established CVD** *(β (95% CI) for Percentage of Panel Meeting Metric Target)*	**p-value****	**Diabetes Screening** *(β (95% CI) for Percentage of Panel Meeting Metric Target)*	**p-value****
Total Daily Time	0.42 (0.06 to 0.78)	0.04	0.45 (0.19 to 0.71)	0.004	0.15 (-0.15 to 0.45)	0.38	-0.01 (-0.32 to 0.30)	0.94	-0.06 (-0.24 to 0.12)	0.71
Time Outside Scheduled Hours	0.66 (0.22 to 1.11)	0.02	0.55 (0.20 to 0.90)	0.007	0.25 (-0.10 to 0.61)	0.34	0.15 (-0.16 to 0.46)	0.94	-0.14 (-0.31 to 0.04)	0.39
Pajama Time	0.24 (-0.14 to 0.63)	0.25	0.002 (-0.0002, 0.005)	0.08	0.20 (-0.04 to 0.45)	0.33	-0.12 (-0.47 to 0.23)	0.94	-0.02 (-0.16 to 0.12)	0.79
Clinical Review Time	0.46 (-0.38 to 1.31)	0.28	0.64 (-0.26 to 1.53)	0.16	-0.74 (-2.13 to 0.64)	0.38	-0.73 (-1.69 to 0.23)	0.84	0.38 (-0.20 to 0.95)	0.40
Notes Time	0.43 (-0.08 to 0.95)	0.15	0.45 (0.06 to 0.85)	0.03	0.23 (-0.28 to 0.73)	0.38	-0.11 (-0.69 to 0.47)	0.94	-0.26 (-0.54 to 0.01)	0.36
In Basket Time	1.89 (0.38 to 3.40)	0.03	1.44 (0.25 to 2.62)	0.03	1.20 (-0.02 to 2.42)	0.30	-0.07 (-1.46 to 1.32)	0.94	-0.23 (-1.07 to 0.61)	0.71

***** Models adjust for PCPs' gender, clinical FTE, average panel risk score, whether PCP's practice is a health center, and hospital (BWH vs. MGH). Standard errors are clustered by PCP practice site.

**p-value reflects adjustment for false discovery rate using Benjamini-Hochberg method

© 2022 Rotenstein LS et al. *JAMA Network Open.*
